# Application of poxvirus K3 ortholog as a positive selection marker for constructing recombinant vaccinia viruses with modified host range

**DOI:** 10.1016/j.mex.2020.100918

**Published:** 2020-05-15

**Authors:** Jingxin Cao, Christine Layne, Jessie Varga, Yvon Deschambault

**Affiliations:** a National Microbiology Laboratory, the Public Health Agency of Canada, 1015 Arlington Street, Winnipeg, Manitoba R3E 3R2, Canada; b Department of Medical Microbiology, University of Manitoba, 543-745 Bannatyne Avenue, Winnipeg, Manitoba R3E 0J9, Canada

**Keywords:** Poxvirus, Vaccinia, Recombination, Host range, k3L

## Abstract

Vaccinia virus is capable of replicating in a wide range of vertebrate animal cells. However, deletion of the two virus host range genes, E3L and K3L, would render replication of the virus abortive in all the cell types tested, except the cells with defective PKR and RNase L activity. By expressing different poxvirus K3 ortholog proteins in the E3L and K3L double knockout vaccinia virus, we can construct a mutant vaccinia virus with modified host range. Here, using poxvirus K3 ortholog as a positive selection marker, we developed a proof-of-concept protocol to construct recombinant vaccinia viruses expressing foreign proteins. Such a recombinant virus has a modified host range in comparison to wild-type vaccinia virus. This protocol offers the following advantages:

➢ Cheap: no additional selection reagent is required.
➢ Highly efficient: nearly all recombinant virus plaques picked would be the stable recombinant virus expressing the protein of interest.
➢ Rapid: starting from transfection with the recombinant DNA vector, a purified recombinant virus expressing the protein of interest could be obtained within a week.

## Graphical abstract

**Figure:**
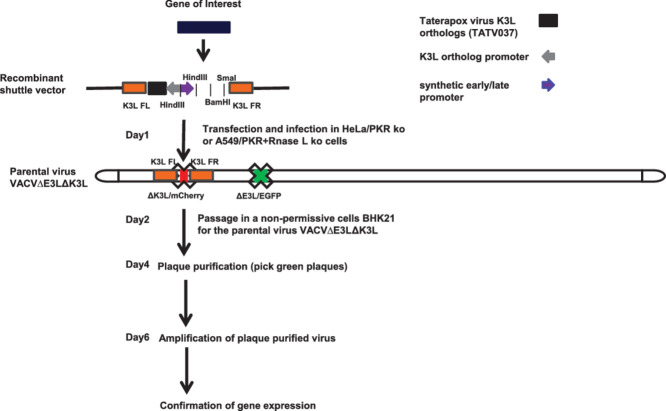

Specifications Table

**Table:** 

Subject area:	Immunology and Microbiology
More specific subject area:	Virology/poxviruses/vaccinia
Method name:	Novel method making recombinant vaccinia viruses
Name and reference of original methods:	N/A
Resources availability:	N/A

## Method details

### Materials

**Fig. 1:**
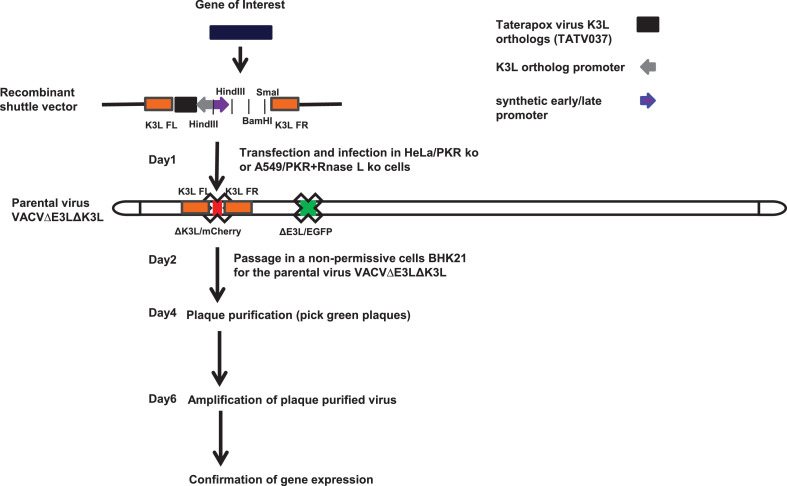
Schematic outline of the method. The components of the recombinant shuttle vector include: the two flanking sequences (K3L FL and K3L FR) which mediate homologous recombination; a poxvirus K3 ortholog gene (the taterapox virus 037 used in this example) driven by a sheeppox virus K3 ortholog promoter; a synthetic early/late promoter; a gene of interest cloned downstream of the early/late promoter; all the components are cloned in the plasmid pUC57. The parental virus for constructing recombinant virus is a vaccinia E3L and K3L double deletion mutant virus, in that the E3L gene is interrupted with an EGFP gene and the K3L is interrupted with a mCherry gene. The parental virus can only replicate in HeLa/PKR knockout (ko) or A549/PKR+RNase L ko cells, in which the homologous replication occurs. The selection and purification of the recombinant are performed in BHK21 cells, in which the parental virus cannot replicate and only the recombinant carrying the taterapox virus K3 ortholog gene can replicate.
➢ Restriction enzymes, T4 DNA ligase, competent *E. coli*, Taq DNA polymerase
➢ Human lung carcinoma cells, A549 PKR and RNase L knockout (ko) cells [5]
➢ HeLa cells, BHK21 cells, HeLa/PKR ko cells [2]
➢ DMEM medium, 10X MEM, fetal calf serum (FCS)
➢ Attractene transfection reagent (Qiagen)
➢ Low melting point (LMP) agarose (Invitrogen)
➢ FLAG antibody (Sigma), vaccinia D12 antibody [6]
➢ Zeiss fluorescence microscope Axiovert 200 M
➢ 12-well tissue culture plates
➢ NUCLISENS easyMAG (BioMerieux)
➢ Primers: K3FLF: AGAGCTCTGATTAGTTGCTGGCAACGA; K3FRR: ACTCGAGTACGTATATTTAGATGTTTTCA; and HHV8K8.1C: TCACACGATATAGGGCTTCTTTCT

➢ Vaccinia Western Reserve strain (WR) E3L and K3L double deletion mutant virus (VACVΔE3LΔK3L) [3]
➢ Recombinant plasmid vector (Fig. 1)

### Procedure

Poxvirus K3 ortholog based selection of recombinant vaccinia virus with modified host range

1. The structure of the recombinant shuttle vector and the parental virus, and the time course of the procedure are outlined in Fig. 1. The recombinant shuttle vector consists of the two flanking sequences matching the corresponding sites in the genome of the parental virus (left flanking sequence nucleotide 27,171–27,305; right flanking sequence nucleotide 27,624–27,971, Genbank accession number NC_006998) used for constructing the recombinant virus. For the proof-of-concept, the K3 ortholog of taterapox virus (TATV037, Genbank accession number NC_008291, nucleotide 26,348–26,614) driven by a sheeppox virus K3 ortholog promoter (Genbank accession number NC_004002, nucleotide 9919–10,250) was placed between the two flanking sequences and used as the host range selection marker. A gene of interest was cloned down stream of the early and late promoter [4] at or between the restriction enzyme sites (*Bam*HI and SmaI). As an alternative, the synthetic early/late promoter can be removed by HindIII digest and replaced with the gene of interest driven by a poxvirus promoter of choice.
2. The general protocol to infect the cells with the parental virus and transfect with the recombinant shuttle vector DNA is similar to the previously described [10]. The following are specific to this procedure: (1) HeLa/PKR knockout (ko) cells or A549/PKR+RNase L ko cells were used in the infection and transfection step; (2) the parental virus used in this protocol must be vaccinia virus E3L and K3L deletion mutant (VACVΔE3LΔK3L, Fig. 1); (3) the cells in a 12-well tissue culture plate were infected with the parental virus at a m.o.i. of 0.1 and transfected with 1 µg of the recombinant shuttle vector; (4) the selection and purification of the recombinant viruses were done in a cell line in which the poxvirus K3 ortholog used would make the parental virus (VACVΔE3LΔK3L) replication competent (see Table 1 in reference [3]); (5) a parental virus infection control should be included.
3. 24 h after infection and transfection (Fig. 2A), the virus was collected by 3 rounds of freeze and thaw and the cell debris was removed by 2 to 3 min centrifugation at 2000 rpm in a benchtop microcentrifuge. 500 µl of a 5-fold serial dilution was added to BHK21 cell monolayers in a 12-well tissue culture plate and incubated at 37 °C, 5% CO_2_ for 1 h. The virus was then removed and replaced with 1 ml of fresh DMEM with 2% FCS, glutamine, penicillin and streptomycin.

**Fig. 2:**
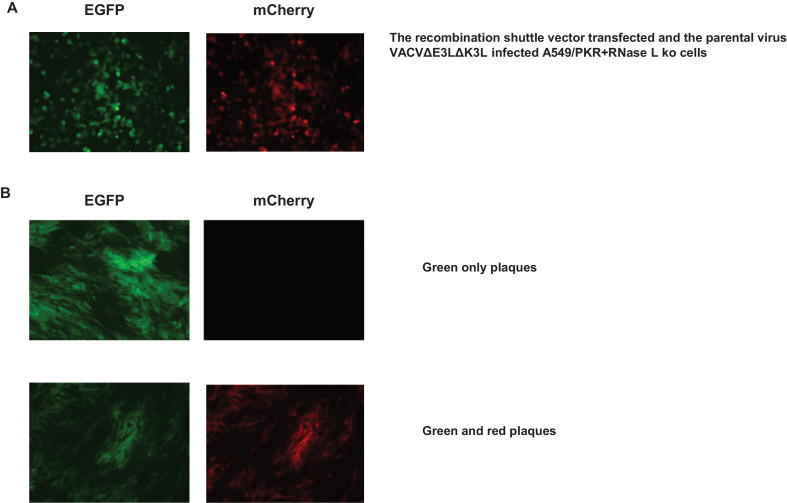
A. Expression of both EGFP and mCherry proteins from the parental virus after infection and transfection in A549/PKR+RNase L kop cells; B Plaque formation 48 h after passage of the collected virus (shown in Fig. 2A) in BHK21 cells; some virus plaques express only EGFP while some virus plaques express both EGFP and mCherry, this passaged virus was further plaque-purified in BHK21 cells.
4. 48 h post infection, the infected cells were examined under a fluorescence microscope. In comparison with the parental virus control, the recombinant virus (collected from HeLa/PKR knockout cells or A549/PKR+RNase L ko cells infected with the parental virus and transfected with the recombinant shuttle vector DNA) should develop noticeable virus plaques as shown in Fig. 2B. Some plaques express only green fluorescent protein, while others express both green and mCherry fluorescent proteins (will be explained in the following paragraph). The cells infected with the highest dilution that developed plaques were collected for further plaque purification in BHK21 cells. At this step, it was not necessary to separate virus plaques expressing different fluorescent proteins.

**Fig. 3:**
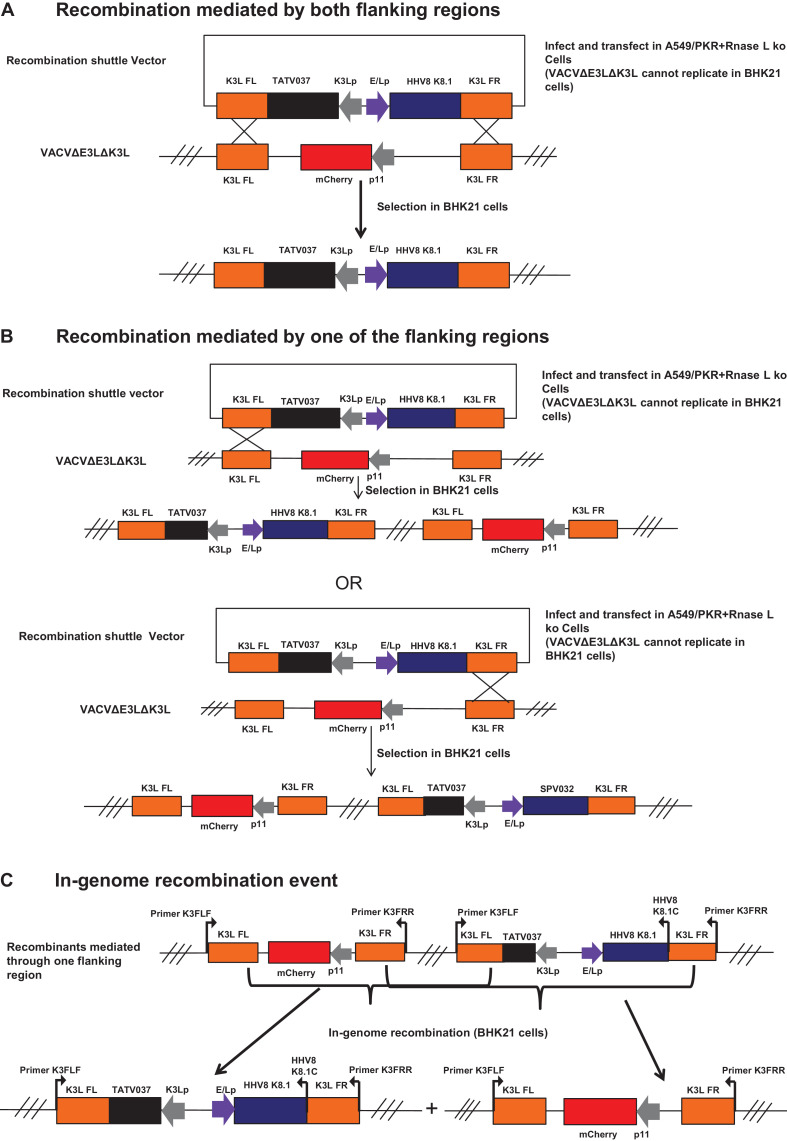
Homologous recombination events between the shuttle vector DNA and the parental virus genome. A: the recombination mediated through flanking left (FL) and flanking right (FR) regions produces a stable recombinant in which the mCherry gene is replaced by the cassette (the taterapox virus K3 ortholog and the gene of interest) from the shuttle vector. B: the recombination mediated through only one of the two flanking sequences, FL or FR, produces recombinants that are not stable since they contain two copies of FL and two copies of FR, and will undergo further in-genome recombination. C: in-genome recombination in which the recombination between the two FL or the two FR regions will generate stable recombinants which contain only one copy of FL and FR.

As shown in Fig. 1, the parental virus (VACVΔE3LΔK3L) expressed EGFP (in E3L locus) and mCherry (in K3L locus) proteins. Two types of recombination events can occur between the recombinant shuttle vector DNA and the parental virus genome. The first type of recombination occurs between the two highlighted regions in the parental virus genome and the corresponding right and left homologous flanking DNA fragments in the shuttle vector (Fig. 3A). This type of event will result in the replacement of the mCherry in the K3L locus by the cassette in the recombinant shuttle vector (the K3 ortholog gene and the gene of the interest). The color of the recombinant virus plaque is only green under a fluorescence microscope. This type of recombinant virus is genetically stable, since there is only one copy of the flanking sequence in the recombinant virus genome. Thus, expressing green fluorescent protein, but not the mCherry, is the indicator of the purity of the selection of the recombinant virus. The second type of recombination occurs between one of the two highlighted regions in the parental virus genome and its corresponding right or left homologous flanking DNA fragment in the shuttle vector (Fig. 3B). This type of recombination will result in the integration of the whole shuttle vector DNA into the parental virus genome. Consequently, the recombinant virus will have two copies of each flanking sequences, the mCherry gene in the parental virus genome will not be replaced by the cassette in the shuttle vector and the virus will produce both EGFP and mCherry proteins. Since this type of recombinant virus has two copies of the identical DNA sequences (one copy from the parental virus and another from the shuttle vector), subsequent in-genome recombination will occur: either to reverse back to the parental virus or to produce the same recombinant virus as the first type of recombination (Fig. 3C).

5. 200 µl of a 5-fold serial dilution of the virus harvested above was added to BHK21 cell monolayers in a 12-well plate. Following 1 h incubation at 37 °C, 5% CO_2_, the virus inoculum was removed, the cell monolayer was washed once with PBS and overlaid with MEM containing 2% FCS, 1x of penicillin-streptomycin-glutamine (diluted from a 100x solution, ThermoFisher), and 1% LMP. The parental virus control was plated out in the same manner. 48 h post infection, the cells were examined under a fluorescence microscope and the plaques which express only green fluorescent protein, but not the mCherry, was marked with a marker, and the virus plaques are picked with a glass Pasteur pipette. In our experience, the purified recombinant virus expressing only the green fluorescent protein can be routinely obtained after 1 round of plaque purification.
6. The plaque-purified recombinant virus is amplified once in BHK21 cells and the virus can be used to infect fresh cells for confirmation of expression of the protein of interest, either by Westernblot or PCR.

### Method validation

Previously, using this selection system we have constructed several recombinant vaccinia viruses expressing genes of various interests: hepatitis E virus capsid protein (for diagnosis of a novel, emerging hepatitis E) [1]; cotton rat CD-40 ligand (for development of research reagent) [9]; and Lassa fever virus glycoprotein (for development of a vaccine candidate, Jingxin Cao unpublished data). Here we used the construction of the recombinant VACV expressing a highly immunogenic human herpesvirus 8 (HHV8) glycoprotein K8.1 (Genbank accession number GU233125) as an example for validating the method.

A FLAG tag was added to the C-terminus of the HHV8 K8.1 gene to facilitate the detection of the protein expression. The tagged HHV8 K8.1 gene was cloned under the control of the synthetic early/late promoter as shown in the Fig. 1. 1 µg of the HHV8 K8.1 shuttle vector was transfected into A549/PKR+RNaseL ko cells, which had been infected with the parental virus VACVΔE3LΔK3L at a m.o.i. of 0.1. 24 h post transfection/infection, the virus was collected and a 5-fold serial dilution of the virus was added to BHK21 cells in a 12-well tissue culture plate. Following 48 h of incubation, the infected cells were examined under a fluorescence microscope. The cells infected with the highest dilution that produced virus plaques were collected and subject to 3 rounds of freeze/thaw. The virus was further plaque purified by adding a series of 5-fold dilution of the virus to BHK21 cells in a 12-well tissue culture plate. Following 1 h incubation, the virus inoculum was removed, cells were washed once with PBS and overlaid with MEM containing 2% FCS, 1x of penicillin-streptomycin-glutamine (diluted from the 100x solution, ThermoFisher), and 1% LMP. 48 h post infection, the infected cells were examined under a fluorescence microscope and the plaques expressing only green fluorescent protein were marked, picked with a glass Pasteur pipette and transferred to a 1.5 ml screw-capped sterile tube. The collected virus plaques were subject to 3 rounds of freeze and thaw and 200 µl of the virus was used to infect fresh BHK21 cells. Following 48 h incubation, the recombinant virus were ready to be collected for confirmation of the expression of the protein of interest, by Western blot and PCR.

**Fig. 4:**
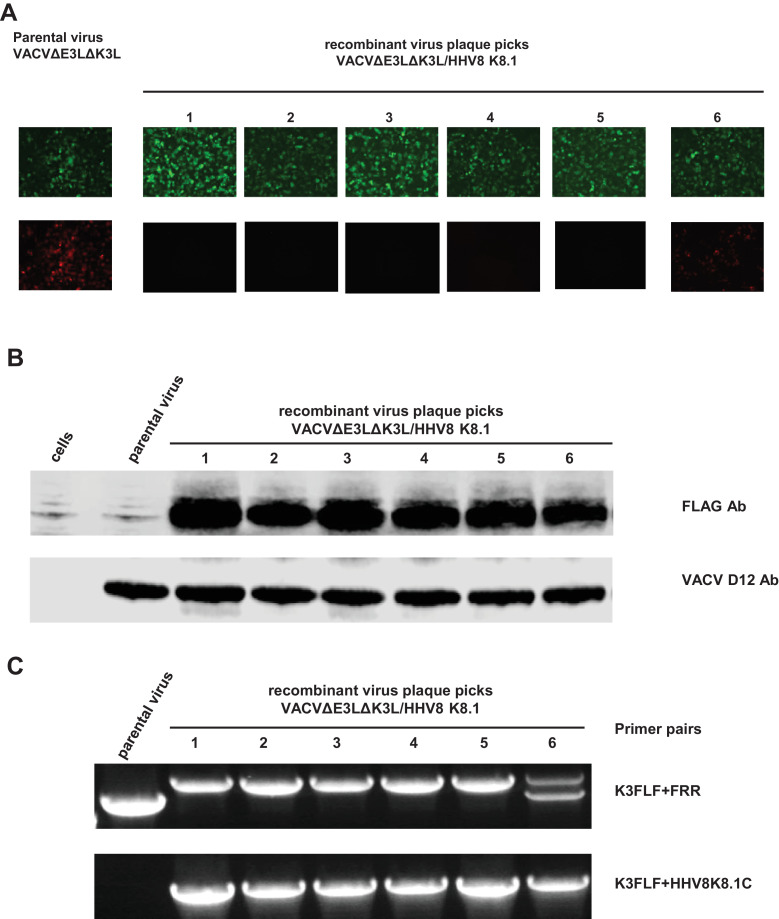
Construction of the recombinant vaccinia virus expressing HHV8 K8.1 protein. A: replication of the recombinant vaccinia virus expressing HHV8 K8.1 protein as shown by expression of EGFP and/or mCherry. The infection was done in A549/PKR+RNase L ko cells in which the expression of the two fluorescent protein could be compared between the parental and the recombinants. B: expression of the HHV8 K8.1 protein shown by Westernblot with a FLAG antibody (tagged to the C-terminus of HHV8 K8.1) with the vaccinia D12 antibody used as a control. The infected cell lysate was from A549/PKR+RNase L ko cells. C: confirmation of insertion of HHV8 K8.1 gene into the recombination locus (vaccinia K3L gene locus) with PCR using virus genomic DNA as template. The primer sites are shown in Fig. 3C.

5 green and 1 red/green plaques (as an example to show it contains a mixture of recombinant viruses) were selected and grown up for examination of the expression of HHV8 K8.1 protein (Fig. 4A). A549/PKR+RNase L ko cells were infected with the selected recombinant viruses and the control parental virus (VACVΔE3LΔK3L) at a m.o.i. of approximately 0.5; the cell lysates were collected at 12 h post infection and subject to Western blot analysis with FLAG antibody and vaccinia D12 antibody. As shown in Fig. 4B, the recombinant viruses from all of the 6 picked virus plaques expressed HHV8 K8.1 protein. However, the protein level from plaque # 6 appeared to be lower than the 5 green only plaques, since the virus from this plaque had a mixed virus population containing the parental virus and the recombinant virus expressing the protein of interest (derived from the single flanking sequence mediated event, Fig. 3B). To further confirm the recombination, the virus DNA was extracted using the NUCLISENS easyMAG (BioMerieux) and PCR with primer pairs, K3FLF+K3FRR and K3L/FLF+K8.1C, was used to examine the integration of the cassette containing HHV8 K8.1 and the K3L ortholog (TATV037). The primer pair K3FLF+K3FRR amplified one DNA product from the recombinant virus DNA extracted from the green only plaques (HHV8 K8.1 and TATV037), one DNA product from the parental virus DNA (mCherry gene), two DNA products from the recombinant virus DNA extracted from the red/green plaque (#6) (mCherry, HHV8 K8.1 and TATV037). The primer pair K3FLF+HHV8K8.1C amplified DNA only from the recombinant viruses and no DNA was amplified from the parental virus DNA (Fig. 3C and 4C).

### Additional comment

Although recombination between the recombinant shuttle vector and the poxvirus DNA genome can readily occur, selection and purification of a recombinant poxvirus can be laborious, mainly due to the recombinant virus accounting for only a tiny proportion in the total virus population. Using the vaccinia E3L and K3L double deletion mutant virus (VACVΔE3LΔK3L) as the parental virus and a shuttle recombinant vector targeting the K3L locus with a poxvirus K3L ortholog as a host range selection marker, this protocol allows the selection and purification of the recombinant virus after one round of amplification and one round of plaque purification. Using this protocol, we can routinely obtain a purified recombinant vaccinia virus within a week starting from the transfection with the recombinant shuttle vectors. Since the parental vaccinia virus used in this protocol does not have the E3L gene, which is critical for the virus pathogenicity, the recombinant virus generated should be highly attenuated. Different poxvirus K3 orthologs have been shown to have different host range activities (see Table 1 in reference [3]) [7,8]. Therefore, using a different poxvirus K3 ortholog (other than the TATV037), a recombinant vaccinia virus with a different host range can be constructed. This feature can be an advantage for certain applications. For example, to make a recombinant vaccinia virus as a candidate vaccine for pigs, we can use the swinepox virus K3 ortholog to construct the recombinant vaccinia virus, which would only infect pigs but not humans. In summary, the method described here has potential for constructing recombinant poxviruses used as vaccine or therapeutic agents.

## Declaration of Competing Interest

The authors confirm that there are no conflicts of interest.
